# Liraglutide versus pramlintide in protecting against cognitive function impairment through affecting PI3K/AKT/GSK-3β/TTBK1 pathway and decreasing Tau hyperphosphorylation in high-fat diet- streptozocin rat model

**DOI:** 10.1007/s00424-024-02933-0

**Published:** 2024-03-27

**Authors:** Hoda M. Moghazy, Nesreen G Abdelhaliem, Sherine Ahmed Mohammed, Asmaa Hassan, Amany Abdelrahman

**Affiliations:** 1 Department of Physiology, Faculty of Medicine, Sohag University, Sohag, 82524 Egypt; 2 Department of Histology, Faculty of Medicine, Sohag University, Sohag, Egypt

**Keywords:** Pramlintide, Liraglutide, GSK-3β, TTBK1

## Abstract

The American Diabetes Association guidelines (2021) confirmed the importance of raising public awareness of diabetes-induced cognitive impairment, highlighting the links between poor glycemic control and cognitive impairment. The characteristic brain lesions of cognitive dysfunction are neurofibrillary tangles (NFT) and senile plaques formed of amyloid-β deposition, glycogen synthase kinase 3 beta (GSK3β), and highly homologous kinase tau tubulin kinase 1 (TTBK1) can phosphorylate Tau proteins at different sites, overexpression of these enzymes produces extensive phosphorylation of Tau proteins making them insoluble and enhance NFT formation, which impairs cognitive functions. The current study aimed to investigate the potential contribution of liraglutide and pramlintide in the prevention of diabetes-induced cognitive dysfunction and their effect on the PI3K/AKT/GSK-3β/TTBK1 pathway in type 2 diabetic (T2D) rat model. T2D was induced by administration of a high-fat diet for 10 weeks, then injection of a single dose of streptozotocin (STZ); treatment was started with either pramlintide (200 μg/kg/day sc) or liraglutide (0.6 mg/kg/day sc) for 6 weeks in addition to the HFD. At the end of the study, cognitive functions were assessed by novel object recognition and T-maze tests. Then, rats were sacrificed for biochemical and histological assessment of the hippocampal tissue. Both pramlintide and liraglutide treatment revealed equally adequate control of diabetes, prevented the decline in memory function, and increased PI3K/AKT expression while decreasing GSK-3β/TTBK1 expression; however, liraglutide significantly decreased the number of Tau positive cells better than pramlintide did. This study confirmed that pramlintide and liraglutide are promising antidiabetic medications that could prevent associated cognitive disorders in different mechanisms.

## Introduction

DM has become the world’s fifth leading cause of death, and the International Diabetes Mellitus Federation estimated that 592 million people will suffer from diabetes mellitus worldwide by 2035 [34].

Cognitive dysfunction (CD) is a form of dementia caused by disrupting the metabolic functions of neurons and glial cells, promoted by the formation of vascular lesions, and sustaining chronic inflammation. Preserving the physiological level of glycemia enhances cognitive functioning, but untreated or improperly managed diabetes increases the risk of dementia. For these reasons, it is crucial to restore adequate glucose metabolism with carefully chosen medication [7]. The American Diabetes Association (ADA) guidelines (2021) place a strong emphasis on the significance of raising public awareness of diabetes-induced cognitive dysfunction, highlighting the links between poor glycemic control and cognitive decline as well as the tendency of chronic diabetes to worsen cognitive function [3].

The characteristic brain lesions of CD are the neurofibrillary tangles (NFTs) and the senile plaques formed of amyloid-β deposition (Aβ), which are features of progressive neurodegenerative disorders [1]. A known risk factor for CD is insulin resistance, which is a common finding in people with T2DM. Abnormal insulin metabolism can hasten cognitive decline by influencing the production and breakdown of Aβ [17]. In general, it is believed that the aggregation of Aβ and hyperphosphorylation of Tau proteins are crucial initiators in the pathogenesis of CD, which results in NFT deposition, neuronal dysfunction, and dementia [10]. The aggregated Aβ activates the proinflammatory response of the microglia, which initiates a neuroinflammatory response in the form of production of the tumor necrotic factor (TNFα), and interleukines like IL1 and IL6 which leads to further damage of neurons, that is why the number of microglia increases in the areas of degeneration and amyloid deposition in brain [21].

Normal insulin signalling is initiated when insulin binds to its receptors with successive activation of the PI3K/AKT pathway, which improves glucose transport and energy metabolism in most tissues of the body including the brain [22]. On the other hand, activation of this pathway leads to inhibition of the glycogen synthase kinase 3 beta (GSK-3), a Tau kinase that promotes deposition of the amyloid-β protein and formation of the NFTs in the neurons.

Insulin resistance is characterized by less sensitive insulin receptors, reduced activity of the PI3K/AKT pathway, and enhanced activity of the GSK-3β, which is in charge of increased tau phosphorylation and augmented amyloid plaque formation, which are the main pathogenic features of CD [2]. As a result, GSK-3b has received most of the attention as an upstream pathogenic promoter for tau hyperphosphorylation and CD [64].

Tau proteins belong to the family of microtubule-associated proteins (MAPs), which are present mainly in the axons of the neurons. Their normal function is to regulate the microtubule assembly, which helps axonal transport, neuronal outgrowth, and polarity [26, 43, 53]. Tau proteins are present in six isoforms inside the neurons and undergo phosphorylation at multiple amino acid residues with different types of kinase enzymes [43]. This is balanced by dephosphorylation by protein phosphatases [20]. Extensive or aberrant tau phosphorylation converts soluble tau proteins into paired helical filaments leading to the development of NFTs, which cause tau pathologies in cognitive dysfunction, Alzheimer’s disease, and other tauopathies.

The highly homologous Tau tubulin kinase 1 (TTBK1) is one of the serine/threonine tyrosine kinases family, It is an important Tau kinase which phosphorylates Tau at certain amino acid residues and has the potential to induce NFTs when overstimulated [53]. *TTBK1 is expressed and acts specifically in neurons* [58]. Its inhibition could relieve disorders like Alzheimer’s disease [65].

The hippocampus is the most important brain structure involved in learning and memory-associated cognitive functions [8]. Unfortunately, the hippocampus is vulnerable to the effects of diabetes [38]. Because all aspects of hippocampal plasticity (i.e., functional and structural synaptic plasticity, and adult neurogenesis) are strongly sensitive to the modulation of insulin signalling in the brain, so changes in the insulin cascade in the hippocampus markedly affect cognitive functions [49].

The incretin hormone glucagon-like peptide-1 (GLP-1) and its synthetic analogue (liraglutide) can decrease blood glucose levels, enhance glucose transport across cell membranes, and decrease insulin resistance.GLP-1, like insulin, acts primarily as a growth factor in the brain that promotes cell growth, proliferation, and repair while inhibiting apoptosis. Liraglutide, a long-acting analogue, can cross the blood–brain barrier (BBB). By boosting long-term potentiation in the hippocampus, facilitating learning, lowering plaque formation and inflammation in the brain, and even boosting neurogenesis, it exhibits physiological effects in the brain [14]. Liraglutide may successfully counteract the negative effects of insulin resistance, making it a potential therapy for CD [24].

The pancreatic peptide hormone; amylin works in conjunction with insulin to reduce blood glucose by slowing gastric and intestinal emptying, inhibiting glucagon secretion, and reducing food intake. The amylin analogue (pramlintide) was approved as a hypoglycemic drug and has recently drawn the attention of researchers studying CD. It easily crosses the BBB and mediates brain processes like increasing neuronal regeneration and boosting glucose metabolism. It is known that amylin interacts with the insulin signalling cascade and stimulates many of insulin’s downstream targets [11]. An amylin analogue called pramlintide was created and is currently being used in clinical settings to treat diabetes. There is currently no information on the impact of pramlintide use on CD induced by type 2 diabetes mellitus (T2DM), even though it is a nontoxic version of amylin that stimulates many metabolic pathways and does not aggregate as amylin also [45].

Whether the GLP-1 or amylin analogues could alleviate diabetes mellitus-induced cognitive dysfunction at cellular and molecular levels is rarely studied in the literature. So this study focused on the preventive effect of these hormone analogues on the expression levels of the GSK-3β, TTBKI, and neurodegeneration and their role in enhancing spacial and long-term memory in a high-fat diet-streptozotocin-induced diabetic rat model.

## Materials and methods

The present study was conducted in the Physiology Department, Faculty of Medicine, Sohag University, and was approved by the Research Ethics Committee considering the care and use of laboratory animals (approval number Sohag- IACUC 5,17,2023,03).

Male Wistar rats (*n* = 60), 7 weeks old and weighing around 85 g, were procured from the Laboratory Animal Center of Sohag University, Faculty of Medicine. Before the experiment began, all rats had a week of acclimatization. Animals have free access to food and water from the tap. At normal ambient temperature and with a typical light/dark cycle, rats were housed in groups in polycarbonate cages (20 × 40 × 20 cm, five rats in each cage). Rats were randomly subdivided into four groups (*n* = 15 in each group).

### Animal groups

**Fig. 1:**
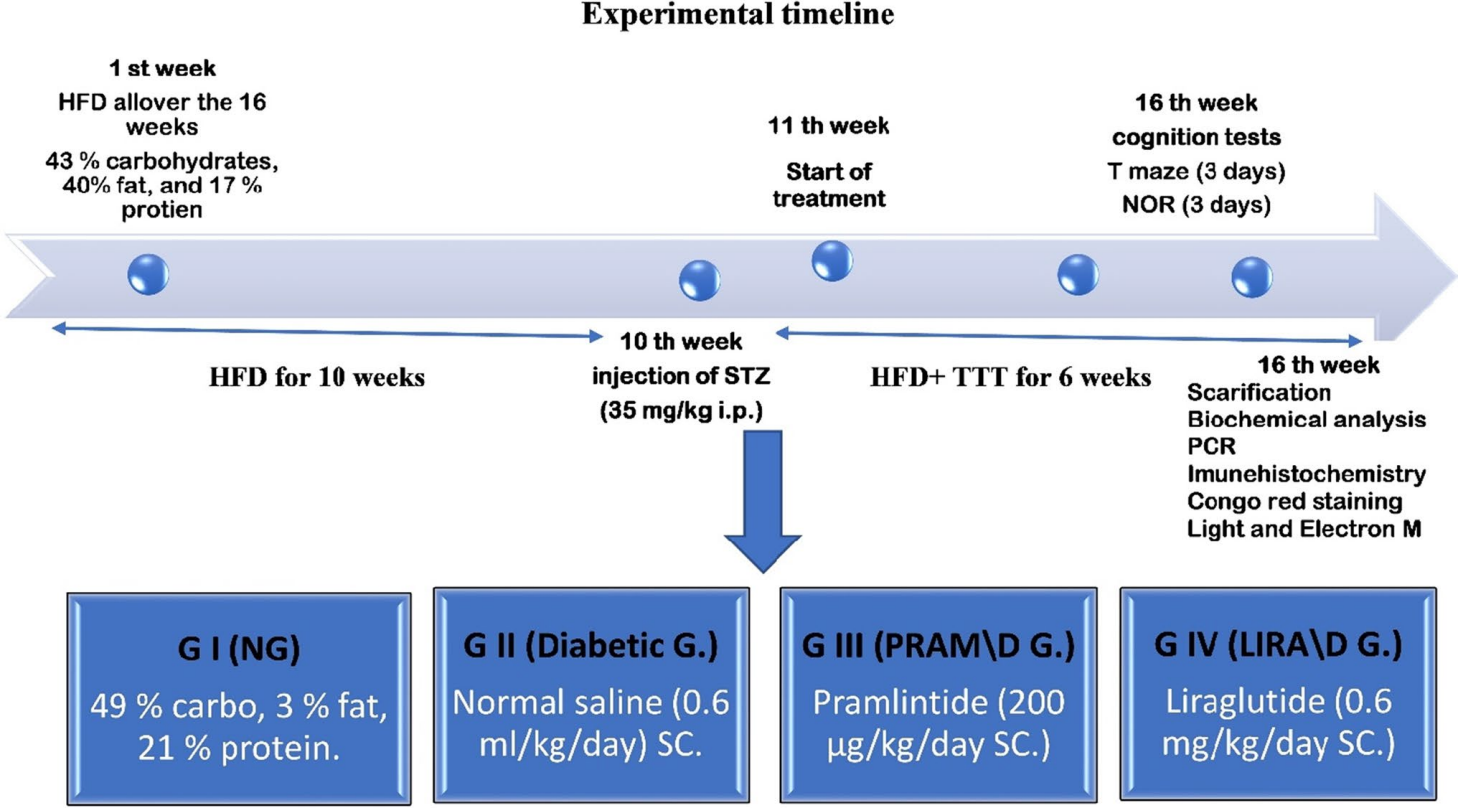
Experimental timeline and animal groups. HFD, high fat diet; STZ, streptozotocin; PRAM, pramlintide; LIRA, liraglutide

The rats were divided into *four groups* (15 rats each); *the control group* was fed normal commercial chow containing 49% carbohydrates, 3% fat, and 21% protein all over the study (16 weeks); *the other groups (2–4)* are the type 2 diabetic model groups that received HFD chow containing 43% carbohydrates, 40% fat, and 17% protein for *10 weeks*, and then type 2 diabetes was induced by a single dose of streptozotocin, STZ (35 mg/kg i.p.) dissolved in 0.1 M citrate buffer (PH 4.5), purchased from Sigma-Aldrich Company (cat. no: 0130). Three days later, blood glucose was measured to confirm the diagnosis of diabetes (fasting glucose level greater than 200 mg/dL is diagnostic) [31]. By the start of the 11th week, rats in *G II; Diabetic G (D G)*, received (0.6 mg/kg/day) of normal saline subcutaneous (SC) injection for 6 weeks. *G III (D\ PRAM G)* rats received pramlintide (200 µg/kg/day SC) for 6 weeks [36]. It was dissolved in 1 ml of DMSO (as a manufactural structure); further dilution of the stock solution was made using 99 ml isotonic saline (PRAM was Purchased from Sigma–Aldrich company cat. No: SML 2523). *G IV (D LIRA\ G)*: rats received liraglutide (0.6 mg/kg/day SC), dissolved in distilled water for 6 weeks [61]. Victoza® (liraglutide) injection was purchased from Novo Nordisk A/S. These groups, G2–4, continued on HFD to the end of the study. Figure 1 explains the experimental design and the animal groups. All rats’ body weight and fasting blood glucose levels (8 h of fasting) were checked every week. Blood glucose is measured by a monitoring device (Bionime GM 300) using a blood drop withdrawn from the rat tail vein.

### Cognitive function evaluation tests

In the 16th week of the experiment, rats were tested for their cognitive functions using the novel object recognition and the T-maze tests.

### T-maze test spontaneous alternation

The spontaneous alternation T-maze is an efficient test for the evaluation of spatial working memory as it relies on the natural behavior of the rodent to explore a new field. T-maze is a polymethylmethacrylate (PMMA) device, formed of a start and two lateral goals (each arm is 50 cm long and 10 cm wide) central choice zone is a square area (10 cm × 10 cm) area. The T-maze is mounted 60 cm above the floor. The procedure steps were done according to [12, 56]. Each rat is tested for five trials. In each trial, the rat is put in the start arm of the T-maze and let to explore the field. When it enters a goal arm whether right or left, the experimenter closes the door of the visited arm for 30 s, then takes out the rat, and puts it in the start arm again, and then if the rat chooses the other arm, this indicates a correct trial, but if it chooses the same visited arm, this is an error trial. The test field is cleaned from any urine or fecal material and then disinfected with 70% ethanol before and between each trial to prevent intra-maze visual and odor cues that could interfere with test reliability. The spontaneous alternation % is calculated as the number of correct choices/5 trial × 100 [12].

### Novel object recognition test

A novel object recognition test (NOR) is a quick and effective method for evaluating memory and learning. The test relies on three sessions: habituation session (T0), training session (T1), and test session (T2). The test had been carried out as described by [6, 30] in an open-field apparatus (50 * 50 *30 cm^3^). At the training session, two identical items (A, A) had been positioned at a constant distance within the apparatus. The contact time with each object was monitored using a stopwatch for 5 min. Twenty-four hours later, the rats were put back in the same field, where the familiar object (A) and a novel object (B) were positioned. Object recognition ability was measured by calculating the discrimination index (DI) which is the time spent exploring—in contact to—the novel object (b) minus the time spent exploring the familiar object (a) divided by total exploration time (DI = B-A/total time), which is unaffected by variations in exploration duration. Also, calculate the recognition index or long-term memory (LTM) as the time of exploration of the new object divided by total exploration time, (LTM = B/total*100).

### Collection of samples

At the end of the experiment, all rats were fasting for 8 h, then anesthetized by 1% inhaled chloroform (0.05 ml/l of container volume [16]. Intra-cardiac blood samples were taken from the heart and then centrifuged at 3000 rpm; serum was preserved at − 20 c until the time of biochemical analysis. After anesthesia, five rats of each group were trans-cardiac perfused with 0.9% saline and then 4% paraformaldehyde in phosphate buffer [55]. The brain was dissected and divided into two parts; the right hippocampus was collected for histopathological evaluation. The other ten rats were decapitated then the brain was dissected and divided into two parts, The right hippocampus was immediately placed in liquid nitrogen and stored at − 80 °C for real-time PCR assay of the genetic expression. In addition, the visceral fat ( mesenteric, epididymal, and retroperitoneal fat pad) was dissected and weighed.

### Biochemical analysis

An ELISA kit was used to determine the serum insulin levels (cat. No: MBS724709, purchased from BioSource company). The following formula was used to estimate insulin resistance using the homeostasis model assessment approach, or HOMA-IR: fasting insulin (IU mg/L) multiplied by fasting glucose (mg/dL) then divided by 405. A commercial ELISA kit was used to quantify serum IL6 to assess the course of the inflammatory response (cat. NO.: EI1006-1, purchased from Assay Pro company).

### Total RNA extraction and real-time PCR

The hippocampus tissue preserved in liquid nitrogen was homogenized. Then the total RNA was extracted, and gene-specific primers were used to detect the PI3K, AKT, GSK-3 β, TTBK-1 protein, and TNF-α activation, with β-actin serving as a housekeeping gene. Thermo-Scientific’s RNA extraction kit (#K0731,0732), Revert aid first strand cDNA synthesis kits (#K1622), and Maxima SYBR Green qPCR master mix (#K0251) were all used according to the manufacturer instructions. Fold expression (2^∆∆CT^) was calculated according to the relative expression of housekeeping gene b-actin. Table 1 shows forward and reverse primer sequences for genes investigated in this study.

**Table 1:** Forward primer and reverse primer for PCR analysis

Gene name	Forward primer	Reverse primer
β actin	ACGGTCAGGTCATCACTATCG	GGCATAGAGGTCTTTACGGATC
PI3K	GCTTCAGGAGGGCCTCGATA	TTTCTTTCAGGGGTCCTATCAGC
AKT	CGCCTGCCCTTCACAACCA	GCCTCTGCTTAGGGTCCTTCTTG
GSK3 β	CTTGGACAAAGGTCTTCCGGCC	GTTGGCAGGCGGTGAAGCAG
TNFα	GTCGTAGCAAACCACCAAGC	TGTGGGTGAGGAGCACATAG
TTBK 1	ACTGAGTACCACACTGCGTC	CGTCCCCAGTGGTGTTAGTG

*PI3K*, phosphatidylinositol 3 kinase; *AKT*, protein kinase B; *GSK3β*, glycogen synthase kinase 3 beta; *TNFα*, tumor necrosis factor α; *TTBK1*, highly homologous kinase tau tubulin kinase 1

### Histological staining

#### Light microscopy

Specimens of the hippocampus after dissection were rinsed in physiological saline and fixed in 10% formalin saline for 24 h. Paraffin wax blocks were sectioned at 5 µm thickness using a microtome (Leica RM 2125) and stained with:

1. Hematoxylin and eosin: for general histological study.
2. Congo red stain: for detection of congophilic amyloid plaque in the hippocampus.
3. Immunohistochemistry staining: for detection of Phospho-Tau-T217 aggregation in the hippocampus (anti-Phospho-Tau-T217 antibody, ABclonal company, China, dilution 1/100, catalog no.: AP1233). Preparation of stained anti-Phospho-Tau-T217 paraffin sections of the hippocampus was done as described by (28). Negative control was done with the omission of primary antibody. Positive control was taken from Alzheimer’s brain tissue (rat cortex and hippocampus). The immunologically positive cells in the hippocampus showed brown cytoplasmic deposits [40].
4. Transmission electron microscopy: Specimens were fixed in 2.5% phosphate-buffered glutaraldehyde and processed by routine protocol. Ultrathin Sects. (75 nm) were stained with 4% uranyl acetate for 20 min and with 0.5% lead citrate for 5 min to be examined by an electron microscope at the Electron Microscopic Unit of Sohag University.

#### Morphometry and statistical analysis

The light microscope Leica ICC50 Wetzlar, Germany at the Histology Department, Faculty of Medicine, Sohag University, was used. Ten nonoverlapping high power fields (×400) for each section in all animals in each group were taken and analyzed using ImageJ 1.51n software (National Institutes of Health USA Java 1.8.0_66 (32-bit) by counting positive cells [23].

Statistical package for the Social Science (SPSS) version 25 was used to analyze the data. Data was expressed as mean ± standard deviation. Multiple comparisons between the experimental groups using one-way ANOVA (Tukey’s test). Statistical significance was considered at a level of *p* < 0.05.

## Results

### Pramlintide and liraglutide significantly decreased body weight and visceral fat in comparison to the diabetic group

**Fig. 2:**
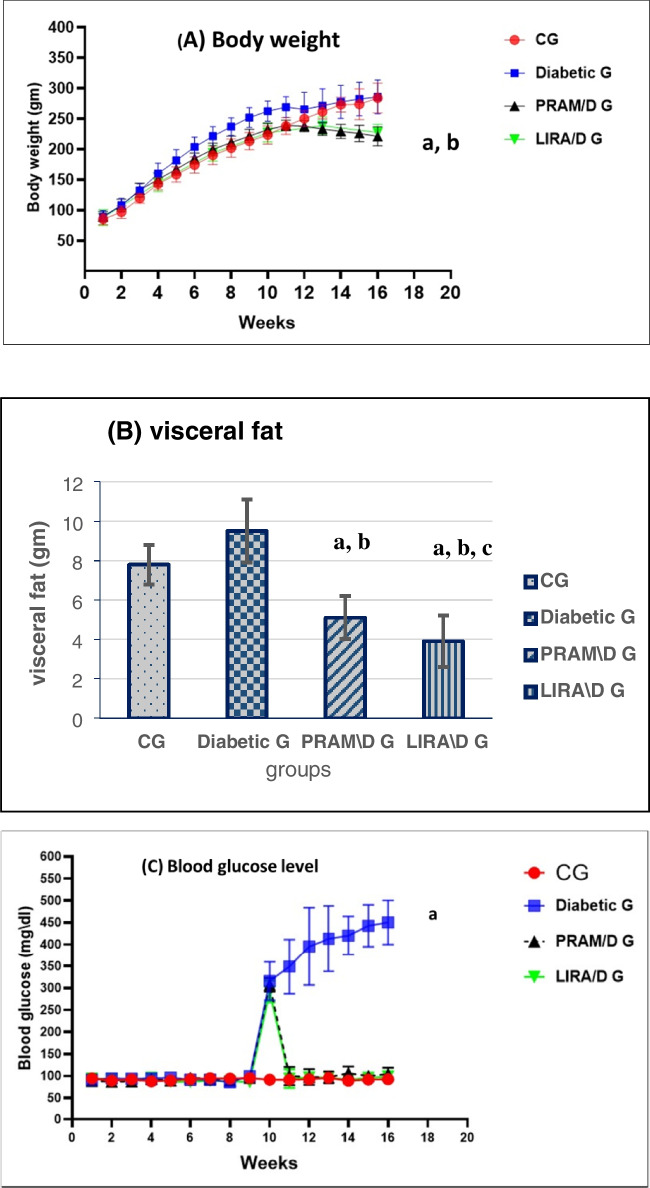
**A** Mean body weight, **B** visceral fat, **C** mean blood glucose level (mg/dl). Data were represented as mean ± SD. Data were analyzed by one-way ANOVA test and post hoc Tukey test (*N* = 15 rats per group). ^a^*p* < 0.05 was significant when compared to CG; ^b^*p* < 0.05 was significant when compared to diabetic G; ^c^*p* < 0.05 was significant when compared to PRAM\D G

By the tenth week of the study, all rats which received HFD had a significant increase in their body weight (*p* < 0.001) in comparison to the CG. During the next 6 weeks of treatment, rats which received PRAM and LIRA showed significantly lower body weight than the DG (0.001). No significant difference in weight was noticed between the PRAM ( 225 ± 15) and LIRA G (227.3 ± 13 *p* = 0.82). In addition, there was no significant difference in weight between the diabetic group and the control group by the end of the study, as we noted a slower rate of growth in the diabetic group than in the control group (as indicated by the flattening in the curve of the diabetic group at the last 3 weeks of the study) which may be related to the catabolic state of the DG due to persistently rising blood sugar (Fig. 2A).

As regards the visceral body fat, there was a significant decrease (*p*-value < 0.001) in the fat pads of the PRAM- (5.9 ± 1.1) and LIRA- (3.8 ± 1.3) treated groups in comparison to both CG (7.8 ± 1.01) and diabetic G.(9.5 ± 1.6), with a significantly lower fat pad in the LIRA G than the PRAM G (*p*-value < 0.01) (Fig. 2B).

### STZ increased BG and insulin levels and HOMA-IR, while pramlintide and liraglutide restored their normal values

Blood glucose level was measured every week during the experiment. A significant increase in BG level at the end of the *tenth week* in all groups was observed as compared to CG (*p* = 0.001) due to induction of DM. at the end of the study, the DG showed still very high blood glucose level (450 ± 46.2) in comparison to the CG (92 ± 11.1, *p* < 0.001). Treatment with either PRAM or LIRA immediately restored normal BG levels as detected by no significant differences between the PRAM and LIRA groups in comparison to the CG (*p* = 0.749, 0.950 respectively), with no significant difference between the PRAM and LIRA groups when compared to each other (*p* = 0.966) (Fig. 2C).

Similarly, the DG showed a significantly *increased insulin* level in comparison to the CG (*p* < 0.001), while treatment with either PRAM or LIRA normalized the serum insulin level as no significant difference between either group compared to the CG (*p* = 0.830, 0.853 respectively). There was no significant difference between the PRAM- and LIRA-treated groups compared to each other (*p* = 0.363) (Table 2).

**Table 2:** Mean insulin level (mg/dl), mean HOMA-IR in different experimental groups

	Normal G (NG) *N* = 15	Diabetic G *N* = 15	PRAM\D G *N* = 15	LIRA\D G *N* = 15	*p*-value
Insulin level (IU mg/L)	10.2 ± 1.8	31.2 ± 3.2^a^	11.04 ± 2.1^b^	9.33 ± 1.9^b^	0.0001
HOMA-IR	2.3 ± 0.33	38.4 ± 4.8^a^	2.8 ± 0.6^b^	2.3 ± 0.5^b^	0.0001
IL 6 level (pg/ml)	2.43 ± 1.9	42.9 ± 12.4^a^	6.6 ± 2.98^b^	3.7 ± 3.5^b^	0.0001

Data were represented as mean ± SD (standard deviation). Data were analyzed by ANOVA test, post hoc, and Tukey test (*N* = 15 rats per group). *p* value was considered significant when *p* < 0.05; ^a^*p* < 0.05 vs. NG; ^b^*p* < 0.05 vs diabetic G; ^c^*p* < 0.05 vs PRAM. G

As expected, the *HOMA-IR* was significantly elevated in the DG (*p* < 0.001 in comparison to CG) but also returned to normal values after PRAM and LIRA treatment (*p* = 0.955, 1.000 respectively in comparison to CG). Again, there is no significant difference between the PRAM and LIRA groups compared to each other (*p* = 0.953) (Table 2).

### STZ increased the serum IL-6 level, while PRAM and LIRA restored its normal level

There was a dramatic decrease in the level of the inflammatory marker, IL-6 level after PRAM and LIRA treatment. PRAM and LIRA groups revealed no significant difference either when compared to CG (*p* = 0.523, 0.973 respectively), or when compared to each other (*p* = 0.782) (Table 2).

### PRAM and LIRA prevented the decline in cognitive function in diabetic rats

Assessment of spatial and long-term memory gave the following results:

### Spontaneous alternation% in T-maze test

**Fig. 3:**
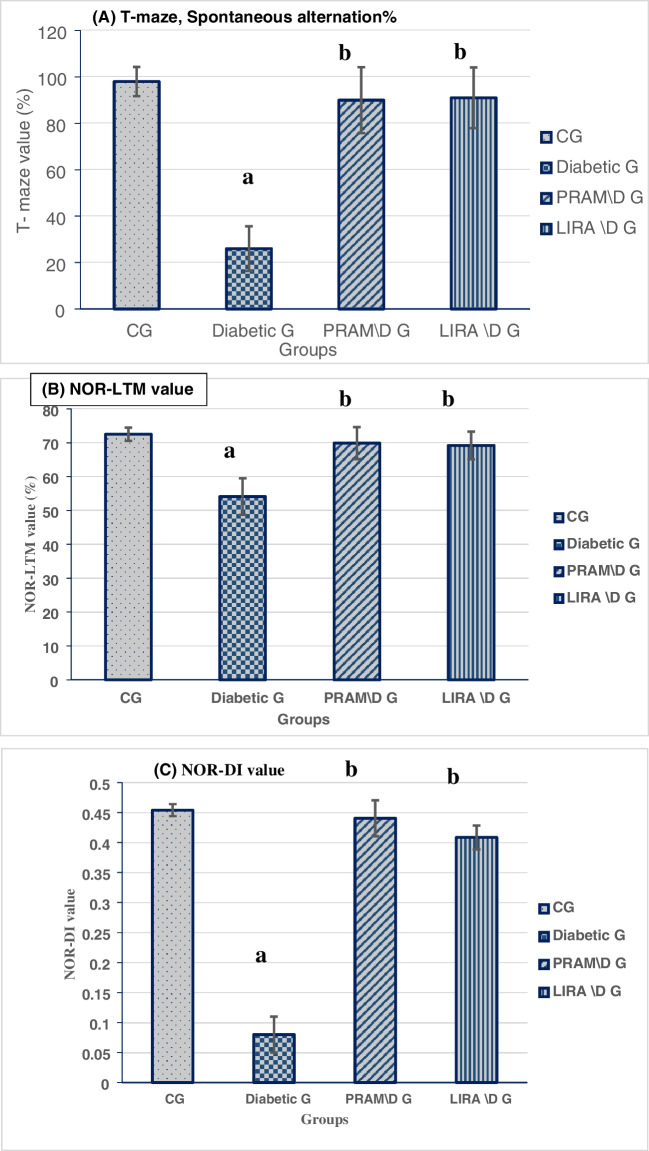
Mean T-maze (**A**) and LTM (**B**) and discrimination index (**C**) results in different groups. Data were represented as mean ± SD. Data were analyzed by one-way ANOVA test and post hoc Tukey test (*N* = 15 rats per group). ^a^*p* < 0.05 was significant when compared to CG; ^b^*p* < 0.05 was significant when compared to diabetic G; ^c^*p* < 0.05 was significant when compared to PRAM\D. G; ^d^*p* < 0.05 was significant when compared to LIRA\DG

The diabetic rats showed impaired spatial memory as shown by significantly decreased alternation % (26 ± 9,6) in the T-maze test when compared to the normal group (98 ± 6.9, *p* < 0.001). However, PRAM and LIRA prevented this severe decline in alternation % and recorded (90 ± 14.1, 91 ± 13.1, *p* < 0.001 compared to the DG). Again, no significant difference between the two drugs (*p* = 1) (Fig. 3A).

### Novel object recognition (long-term memory)

Again, diabetes significantly decreased long-term memory (54.1 ± 5.4) as compared to the normal group (72.5 ± 1.96, *p* < 0.001). However, PRAM and LIRA prevented the decline in the long-term memory results (69.9 ± 4.7, 69.2 ± 4.1, *p* < 0.001 compared to the DG). Noticeably, the LIRA group and PRAM group revealed no significance as compared to each other (*p* = 0.997) (Fig. 3B).

### Novel object recognition (discrimination index)

The discrimination index decreased significantly in the DG (0.08 ± 0.03 vs 0.45 ± 0.01 for the CG, *p* < 0.001). This index was increased to 0.44 ± 0.03 and 0.4 ± 0.02 by PRAM and LIRA treatment respectively. No significant difference was noticed between the PRAM and LIRA when compared to the CG (*p* = 0.507, 0.829 respectively), nor when compared to each other (*p* = 0.9) (Fig. 3C).

### Quantitative real-time PCR (qRT-PCR) analysis

#### The expression level of PI3K and AKT

The diabetic rats showed significantly decreased expression of both PI3K and AKT genes, which reflects decreased tissue sensitivity to insulin, in comparison to the control group *p* < 0.001 for both genes. 

**Fig. 4:**
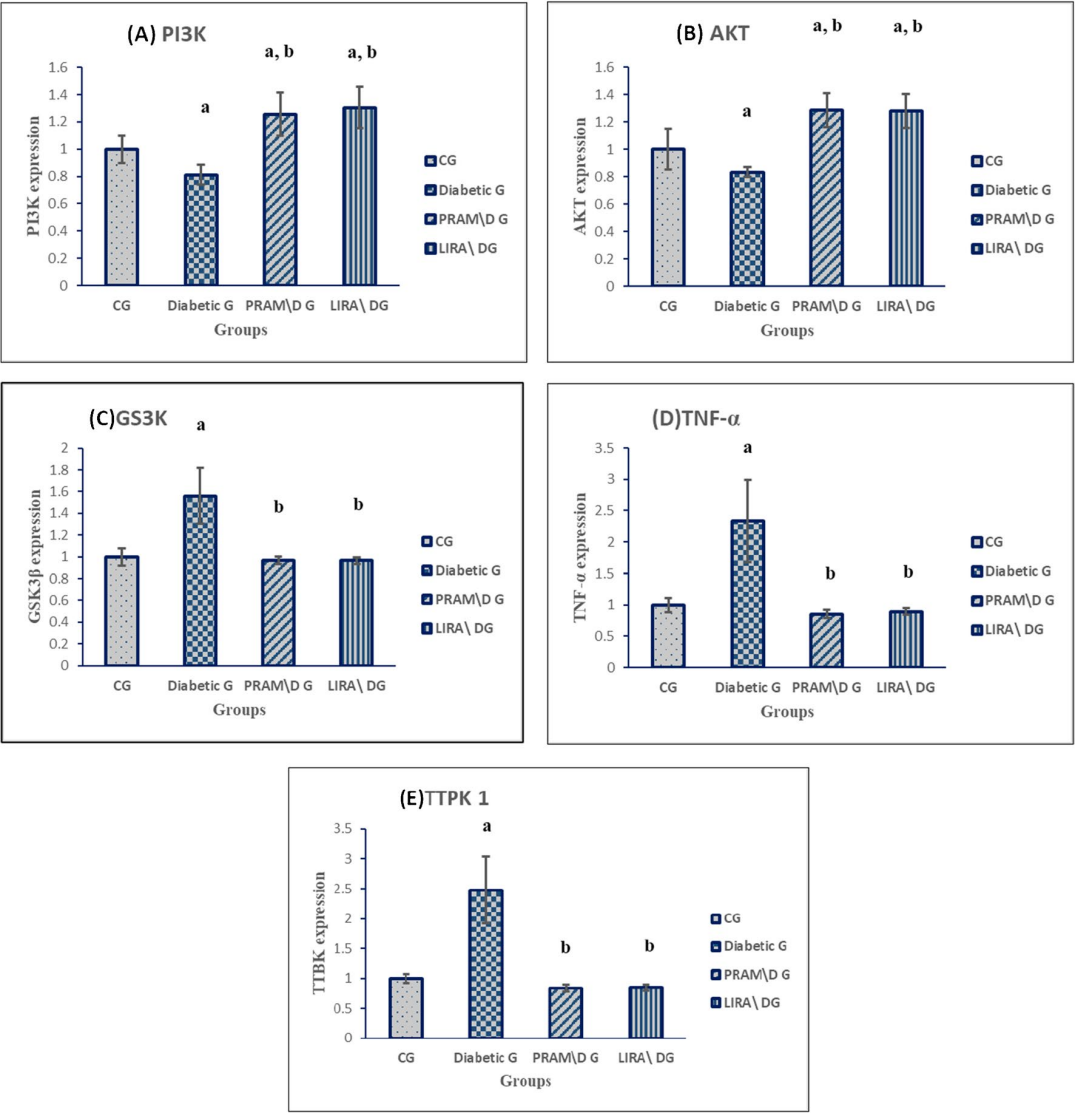
**A** Mean expression level of PI3K, **B** AKT, **C** GSK3β, **D** TNFα, **E** TTBK1. Data were represented as mean ± SD. Data were analyzed by one-way ANOVA test and post hoc Tukey test (*N* = 10 rats per group). ^a^*p* < 0.05 was significant when compared to NG; ^b^*p* < 0.05 was significant when compared to diabetic G; ^c^*p* < 0.05 was significant when compared to PRAM. G

A noticed overexpression of PI3K gene in both LIRA group (1.3 ± 0.15) and PRAM group (1.25 ± 0.16) as compared to the diabetic group (*p* < 0.0001), with no significant difference between the PRAM group and LIRA group compared to each other (*p* = 0.791) (Fig. 4A).

An overexpression of the AKT gene was noticed in both the LIRA group (1.28 ± 0.12) and PRAM group (1.29 ± 0.13), as compared to the diabetic group (0.83 ± 0.04) (*p* < 0.001). LIRA and PRAM groups revealed no significant difference in gene expression when compared to each other (*p* = 0.999) (Fig. 4B).

### The expression level of GSK3 β

There was a noticed overexpression of the GSK3 β gene in the diabetic group (1.6 ± 0.26) as compared to CG (*p* < 0.001). A significant downregulation of the GSK3 β gene in the PRAM group (0.97 ± 0.03) and LIRA group (0.96 ± 0.03) as compared to the DG (*p* < 0.0001). Also, the LIRA group showed no significant difference in gene expression compared to the PRAM group (*p* = 1) (Fig. 4C).

### The expression level of TNFα

There was a significant increase in TNF-α expression in the diabetic group (2.3 ± 0.65) as compared to CG (*p* < 0.001). Noticeably, both LIRA (0.9 ± 0.05) and PRAM (0.85 ± 0.07) groups showed a significant decrease in gene expression compared to the DG (*p* < 0.001), with no significant difference when compared to each other (*p* = 0.991). (Fig. 4D).

### The expression level of TTBK 1

There was a noticeable increase in TTBK1 expression in the diabetic group (2.5 ± 0.6) as compared to CG (*p* < 0.001). LIRA group (0.85 ± 0.05) and PRAM group (0.84 ± 0.06) showed a significant decrease in TTBK 1 gene expression as compared to the DG (*p* < 0.001). Moreover, LIRA and PRAM groups showed no significant change in gene expression when compared to each other (*p* = 1) (Fig. 4E).

### Histological assessment

#### H&E and electron microscopic examination

**Fig. 5:**
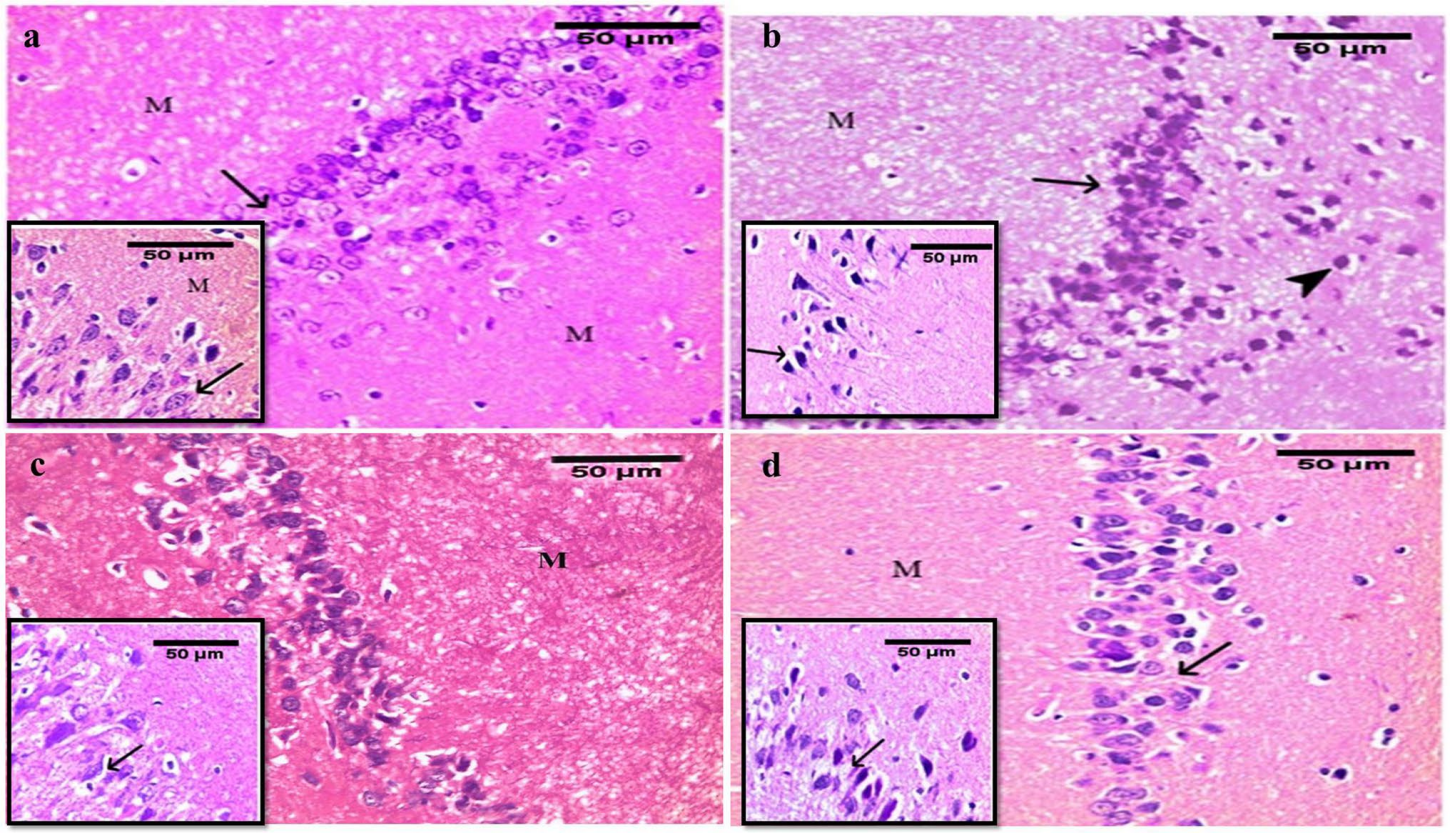
Photomicrographs of the hippocampus in albino rat: **a** control group showing 5 to 6 compact layers of small pyramidal cells (arrow) in the CA2 region, with uniform size and evenly arranged with thin neuropil in between. Each neuron has a rounded central vesicular nucleus with prominent nucleolus and basophilic cytoplasmic stippling. The inset shows a few layers of large pyramidal cells (arrow) in the CA3 region. **b** Diabetic group showing decreased thickness of small pyramidal. Many have pyknotic nuclei and acidophilic cytoplasm (arrow). The inset shows layers of large pyramidal cells showing shrinkage of many cells mainly in the outer layer having hyperchromatic nuclei (arrow). **c** The PRAM-treated group showing preservation of the thickness of small pyramidal layers. The inset shows large pyramidal cells that have basophilic cytoplasm and vesicular nuclei with less apoptotic cells (arrow). **d** The LIRA-treated group was more or less similar to the control group normal. The inset shows a few apoptotic large pyramidal cells with pyknotic nuclei and acidophilic cytoplasm (arrow). Molecular layer (M). (H&E × 400, bar = 50)

**Fig. 6:**
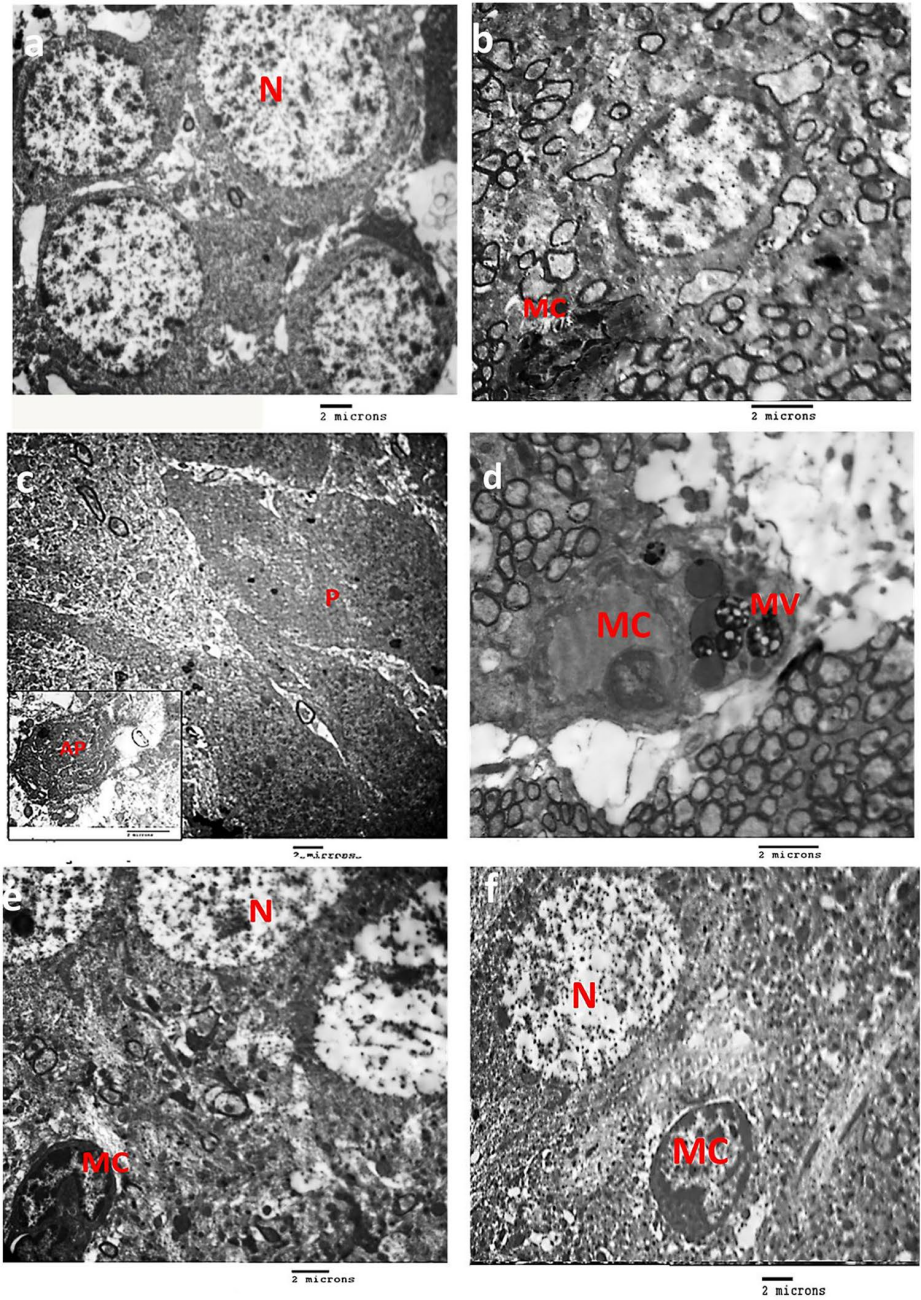
Electro-micrographs of the ultrathin section in the hippocampus albino rat: **a** the control group showing pyramidal cells with a large rounded euchromatic nucleus (N). **b** The control group showing microglial cells (MC) with indented nucleus and dense cytoplasm **c** The diabetic group showing pyramidal cells with apoptotic changes with irregular dense nucleus (P). The inset shows an apoptotic body (AP). **d** The diabetic group shows a microglial cell (MC) with an indented nucleus and multivesicular bodies (MV). **e** The PRAM group and **f** LIRA group showing pyramidal cells with rounded euchromatic nucleus (N) and microglial cell (MC) with an indented nucleus and dense cytoplasm (**a**, **c**, **e**, **f** 3600 × , **b**, **d** 5800 × , inset 10,000 × bar = 2 µm)

In CG, histological examination revealed multiple compact layers of small pyramidal cells of the CA2 region. They were uniform in size and evenly arranged with a thin neuropil in between. They had a rounded central vesicular nucleus with prominent nucleolus and basophilic stippling in the cytoplasm. The molecular layer had glial cells among the neuronal processes. The inset showed large pyramidal cells in the CA3 region (Fig. 5a). Electron microscopic examination of the ultrathin sections of the CA3 area obtained from the control animals revealed its normal electron microscopic picture (Fig. 6a); the microglia shows an indented nucleus and electron-dense cytoplasm (Fig. 6b). In diabetic G, the analysis revealed disorganization with a decreased thickness of small pyramidal cells up to three layers in some areas. Many apoptotic pyramidal cells with pyknotic nuclei and dark acidophilic cytoplasm were frequently seen. The molecular layer contained some apoptotic cells. The inset showed shrinkage of large pyramidal cells with hyperchromatic nuclei (Fig. 5b). On electron microscopic examination of the diabetic group, most pyramidal cells showed signs of apoptosis: shrunken cells with an irregular heterochromatic nucleus and electron-dense cytoplasm. The inset showed an apoptotic body in (Fig. 6c). Moreover, the diabetic group shows a microglial cell with an indented nucleus and multivesicular bodies (Fig. 6d). Upon examination of PRAM\D G, an improvement was noticed compared to the diabetic group with the preservation of the small pyramidal layer thickness with some empty areas seen. The neurons had vesicular nuclei with prominent nucleoli. Also, a few cells have pyknotic nuclei. The molecular layer was seen as more or less normal. The inset square showed many large pyramidal cells had vesicular nuclei in CA3 (Fig. 5c). In LIRA\D G, examination revealed the preservation of small pyramidal layers. The neurons were heavily crowded with thin neuropil in between. The inset showed that most of the cells were more or less like the CN (Fig. 5d). Electron microscopic examination of both PRAM group and LIRA group pyramidal cells appeared more or less similar to the control. Microglia had heterochromatic indented nuclei (Fig. 6e, f).

#### Congo red staining (amyloid β plaques examination)

**Fig. 7:**
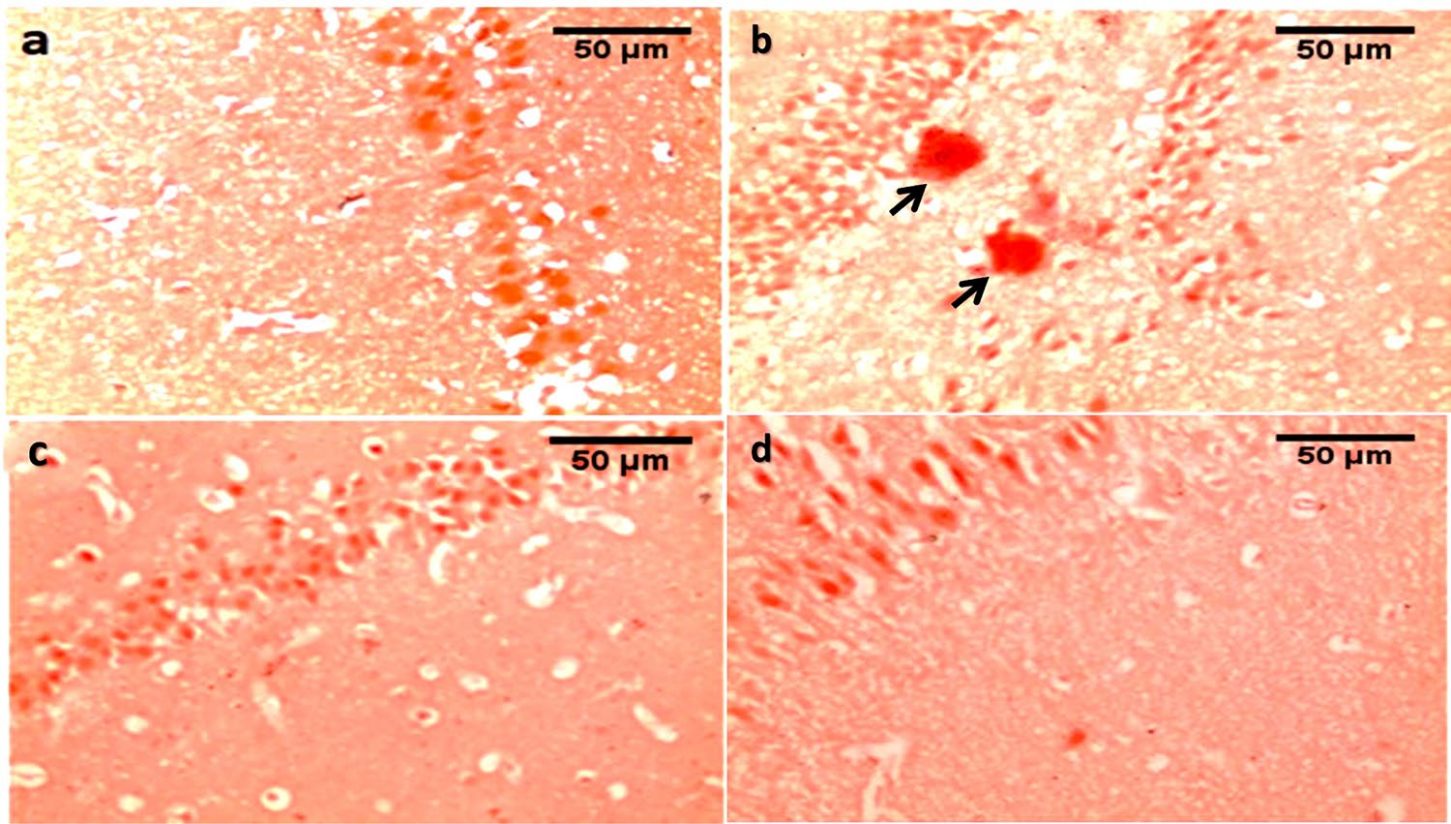
Photomicrographs of Congo red stained hippocampus tissue showing **a** control group: Hippocampus: no amyloid β plaques. **b** Diabetic group: showing large, multifocal deposition of amyloid β plaques (arrow). **c** Pramlintide group: showing no amyloid β plaques. **d** Liraglutide group: showing no amyloid β plaques. (400 × , bar = 50µm)

CG showed no amyloid β plaques deposition between the neuronal cells (Fig. 7a). Upon examination of the diabetic G, the hippocampus showed a large, multifocal deposition of amyloid β plaques (Fig. 7b). In PRAM\D G, hippocampus showed no amyloid β plaques (Fig. 7c). Moreover, the hippocampus showed no amyloid β plaques in the LIRA\D G (Fig. 7d).

#### Immunohistochemistry examination phospho-tau (t-217)

**Fig. 8:**
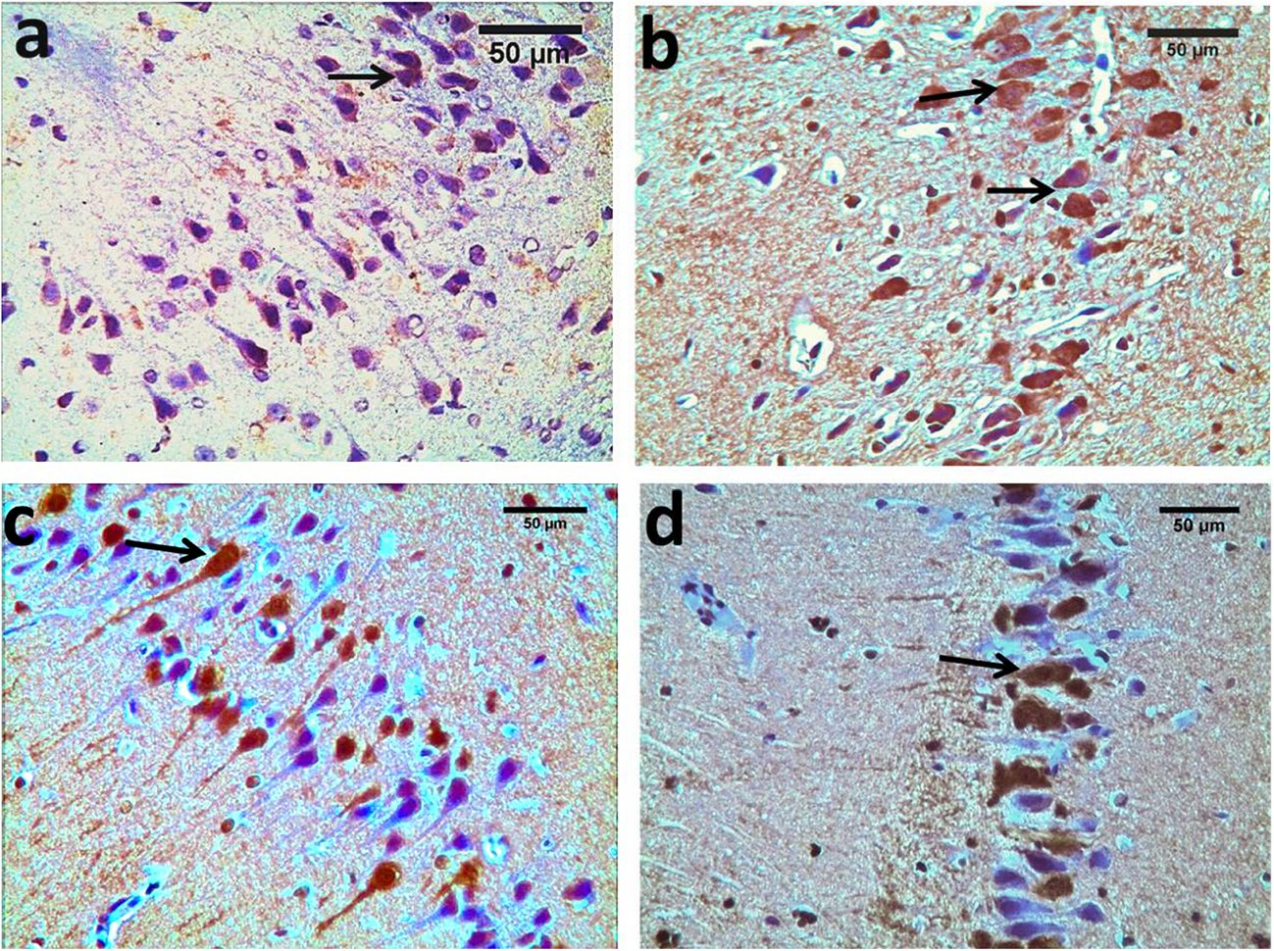
Photomicrographs of rat hippocampus sections in different groups. **a** control, **b** diabetic group, **c** pramlintide group, **d** liraglutide group. In the control, faint cytoplasmic staining was observed in a few pyramidal cells (**a**). Group II: numerous positive pyramidal cells for phospho-tau than the control group (**b**). The pramlintide and liraglutide groups: positive pyramidal cells decreased as compared to control (**c**, **d**). Positive cells (arrow) (Phospho-tau 217 antibody 400 × , bar = 50µm)

**Fig. 9:**
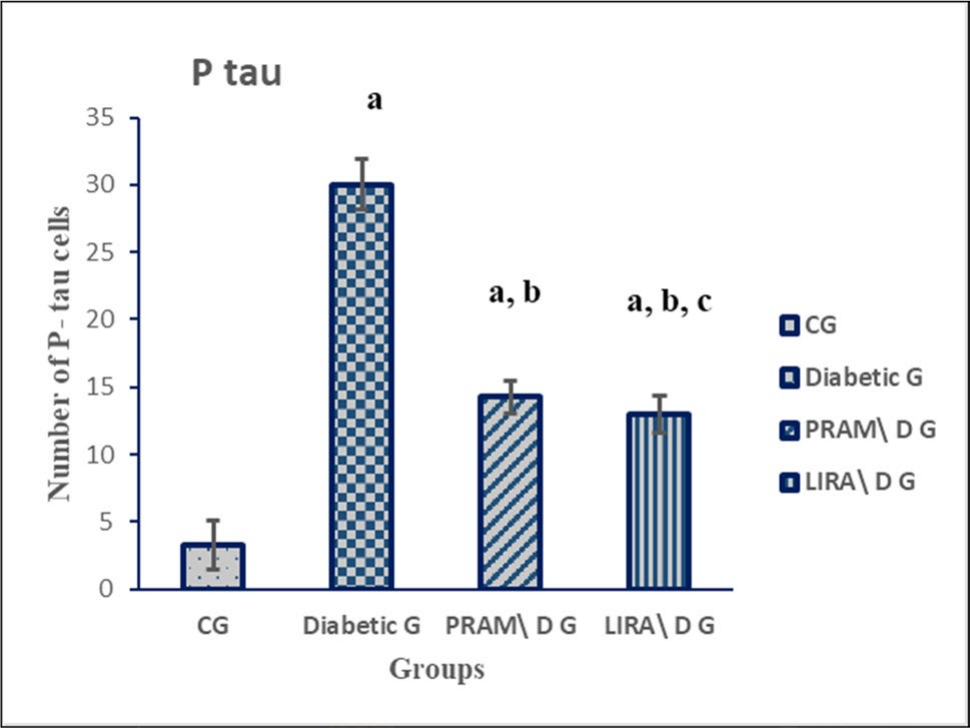
Mean of number of P-tau positive cells. Data are represented as mean ± SD (standard deviation). Data were analyzed by ANOVA test, post hoc, and Tukey test (*N* = 5 rats per group). *p* value was considered significant when *p* < 0.05. ^a^*p* < 0.05 vs. NG; ^b^*p* < 0.05 vs diabetic G; ^c^*p* < 0.05 vs PRAM. G

In control (CG), faint positive brown cytoplasmic staining was observed in a few pyramidal cells (Fig. 8a). In the diabetic group, there were numerous positive immunostaining cells with a significant increase in the number of phospho-tau positive pyramidal cells in CA3 as compared to the control group (Fig. 8b). However, in PRAM and LIRA groups, there was marked attenuation of phospho-tau expression compared with the diabetic group (Fig. 8c, d). A significant increase in phospho-tau immune expression was seen in the LIRA group and PRAM group when compared to CG (*p* = 0.001). Moreover, there was a significant difference between groups PRAM group and LIRA group (*p* value = 0.03). Both treated groups, the LIRA group and the PRAM group, showed a significant decrease in phospho-tau immune expression compared to the diabetic group (*p* = 0.001) (Fig. 9).

## Discussion

**Fig. 10:**
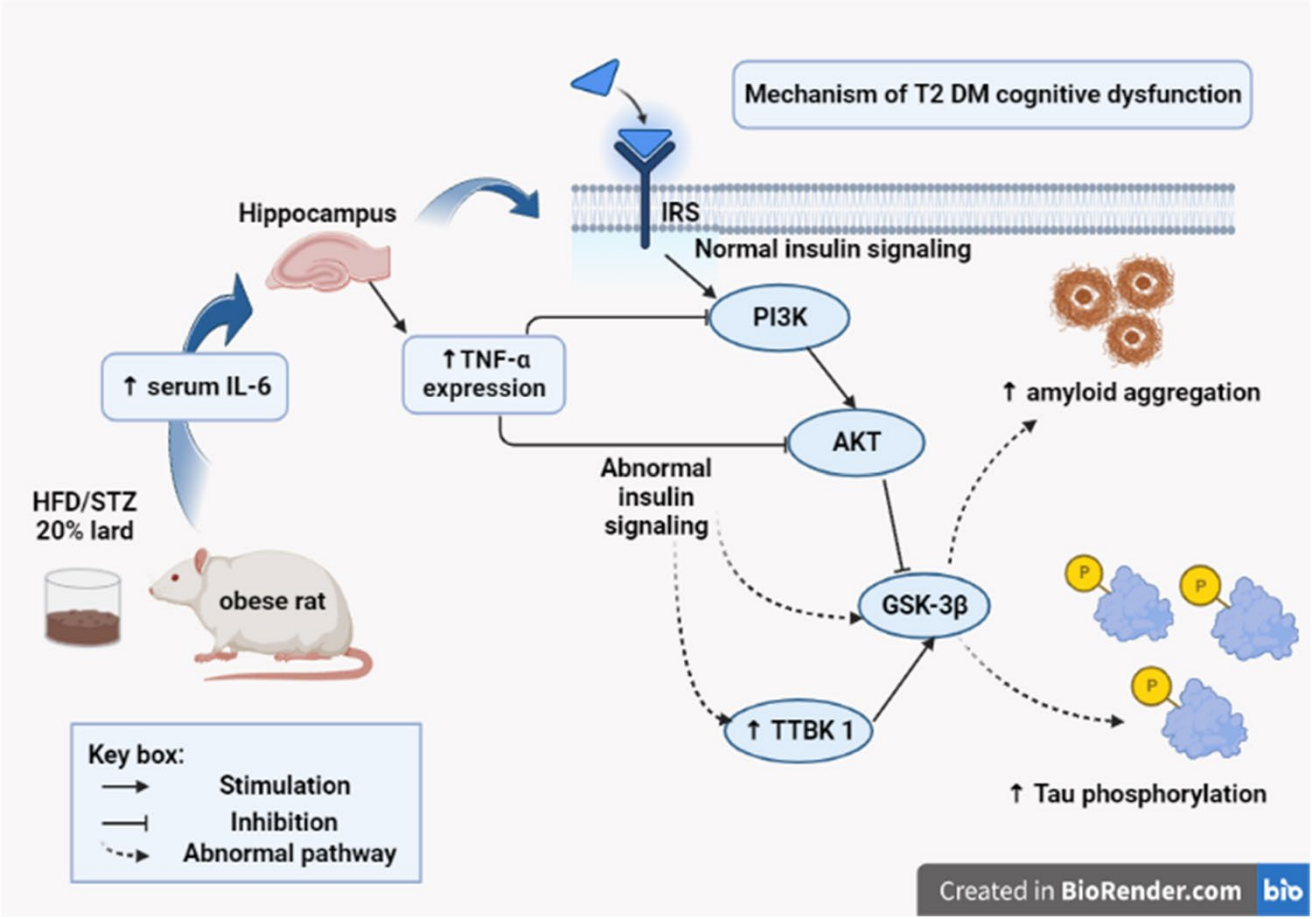
Mechanism of T2DM cognitive dysfunction; unhealthy high-fat diet food associated with increased inflammatory mediators (IL-6) in the circulation with increased TNF-α brain expression. This mainly led to abnormal brain insulin signalling with decreased activity of PI3K/AKT and increasing the activity of TTBK-1 and GSK-3β, which leads to increased amyloid aggregation and increased tau phosphorylation. This potentiates the cognitive disorder associated with T2DM

Several studies found an obvious link between T2DM and the development of cognitive dysfunction [42]; they might share some pathophysiological features, such as abnormal insulin signalling [7] and inflammatory reactions [5]. This work provides a molecular perspective on the potential mechanisms of action of the liraglutide and pramlintide on cognition and insulin sensitivity in an HFD/STZ-induced T2DM male rat model (see Fig. 10 for the pathophysiology of CD in T2D).

In the current study, T2DM was induced by administration of HFD for 10 weeks followed by a single dose, I.P. injection of STZ (35 mg/kg). This model was chosen because HFD led to marked obesity, In addition, low-dose STZ injection caused mild dysfunction in β-cells of the pancreatic islets without completely compromising insulin secretion. This model was done by many researchers who found that this combination of HFD and low-dose STZ injection mimics the chronic onset of T2DM with high hyperinsulinemia [13, 18, 56].

This work demonstrated that HFD/STZ produced significantly high levels of blood glucose and serum insulin with elevated HOMA-IR results compared to the CG. These results are in line with [13, 18]. On the other hand, either PRAM- or LIRA-treated groups showed normal blood, glucose, insulin, and decreased HOMA-IR levels. These results are in line with many researchers who declared that both LIRA and PRAM are new effective treatments for T2DM [11, 36, 59].

This HFD/STZ model produced significant deterioration in cognitive functions as manifested by impaired spontaneous alternation % and recognition index versus those in CG. This was in agreement with the study of Wang et al., who postulated that old diabetic rats displayed reduced spatial learning and short- and long-term memory [52]. Treatment with either liraglutide [47] or pramlintide [9] resulted in more or less normal results of spontaneous T maze as well as NOR test in both treated groups as compared to the diabetic group.

The functional cognitive abnormalities noticed in the diabetic group in this work were confirmed by structural hippocampal histopathological aberrations in the form of neurodegeneration seen mainly in CA2 and CA3, with a decrease in the thickness of the pyramidal cells in CA3, and multivesicular bodies were observed in activated microglia as examined by H&E. This came in agreement with previous studies that showed hippocampal neurodegeneration [4, 57] and multivesicular bodies in activated microglia [4] in diabetic rats. We further confirmed these results by the electron microscopic (EM) findings that showed many apoptotic bodies and shrunk cells with irregular hyperchromatic nuclei with dense cytoplasm. Our ultrastructure findings were consistent with other studies [15, 44, 60].

The PRAM- and LIRA-treated diabetic groups showed improved histological pictures of the hippocampus by the light and EM examination. The thickness of the small pyramidal layers was preserved. Most of the pyramidal cells were more or less normal. The microglial cells showed no histological signs of activity. Other studies discussed how PRAM might improve cognitive function considering the importance of the native role of amylin in the regulation of oxidative processes and neuronal function [39]. Anti-apoptotic properties and oxidative stress reduction of PRAM and LIRA therapy seem to contribute to neurological recovery [11, 28, 47, 62]. A similar finding was observed by Patrick et al., who stated that amylin treatment revealed restored lost mitochondrial membrane potential [39].

Insulin signalling markers in this study significantly deteriorated in the diabetic group versus CG. There was a significant decrease in PI3K/AKT expression, with a significant GSK-3 β and TTBK1 overexpression in the hippocampus. This was supported by the results of Akhtar et al., which showed that there were decreased gene expressions of insulin signalling molecules such as IRS-1, PI3K, and AKT and increased gene expression of GSK-3β in liver cells of diabetic patients [2]. Further studies supported that the kinase TTBK1 had been associated with neurological disorders and is primarily expressed in the central nervous system [33, 37].

On the other hand, both PRAM- or LIRA-treated groups showed an improvement in the brain insulin signalling markers in the form of increased expression of PI3K/Akt and decreased expression of GSK-3 β and TTBK1 in both treated groups versus the diabetic group. To our knowledge, this research is one of the few articles that discussed the relation between the amylin analogue pramlintide and brain GSK-3β expression in cognitive disorders associated with T2DM. Yao et al. demonstrated that liraglutide exerted a neuroprotective effect by activating the PI3K/Akt/GSK3β signalling pathway [59]. Also, Jantrapirom et al. supposed that liraglutide could rescue tau hyperphosphorylation, by sensitizing neuronal insulin signalling [24].

In the present work, Congo red staining examination revealed multifocal deposition of amyloid β plaques in the hippocampus of the diabetic group. Further, immunohistochemical examination of the hippocampus in this study revealed overexpression of Phospho-Tau-T217 in the diabetic group versus the CG. Khalil et. and Setti et al. declared that compromised insulin signalling causes amyloid β peptide accumulation in the brain [25, 46]. Interestingly, treatment with either liraglutide or pramlintide in our study showed a reduction in Aβ plaque burden in the hippocampus with Congo red stain and attenuation of p-tau217 expression with immunohistochemical examination as compared to the diabetic group. These findings support that improvement in neuronal insulin signalling pathways leads to a decrease in tau hyperphosphorylation [54] Our results follow studies done by other researchers which confirmed that treatment with oral amylin lessens amyloid pathology aggregated p-tau and neuroinflammation seen in the CD brain [27, 35].

The present study provides evidence supporting the idea that hyperinsulinemic condition causes neuronal insulin resistance, as indicated by the downregulation of insulin signalling molecules, including PI3K/AKT, with the upregulation of GSK-3β and TTBK1. AKT usually acts as an inhibitor of GSK by inducing the phosphorylation of N-terminal serine 21 of GSK-3α and serine 9 of GSK-3β, thus diminishing prime-substrate phosphorylation by GSK-3 [19]. GSK-3β, a major tau kinase, has been shown to induce abnormal tau hyperphosphorylation [32]. Moreover, TTBK1 might be involved in the initial stages of tau phosphorylation and pre-tangle formation in cognitive disorders pathology [50]. Tau hyperphosphorylation results in an instability of microtubules, which causes the formation of neurofibrillary tangles (NFTs), one of the histopathological hallmarks of cognitive disorders [54]. Also, Toral-Rios et al. reported that normal insulin signalling could reduce tau phosphorylation by inhibiting GSK-3β function through activating the PI3K/Akt pathway. Insulin gene knockout mice were found to show an increase in GSK-3β activity and tau hyperphosphorylation, with excessive deposition of amyloid plaques in hippocampal neurons [4].

Furthermore, several studies have reported that insulin resistance can promote the onset of cognitive disorders by enhancing pro-inflammatory cytokines [49]. Our study supported this hypothesis. This was represented by the significant increase in the serum level of IL-6 and hippocampal tissue expression of TNF-α in the diabetic group as compared to the CG. Akhtar et al. stated that neuroinflammation was the core feature of neurodegeneration [2]. Moreover, TNF-α disrupted neuronal mitophagy, increased oxidative stress, triggered inflammation, dysregulated GSK-3 β activity, and promoted p-Tau accumulation [63]. Further, other studies stated that neurodegeneration in patients with T2DM could be considerably lowered by targeting cytokines, cytokine receptors, and nuclear transcription factors linked to inflammation, like IL-6 and TNF- α, alone or in combination [48]. In this work, pramlintide and liraglutide exhibited anti-inflammatory effects through significantly decreasing the inflammation markers: serum IL-6 and hippocampal TNFα expression when compared to the diabetic group. Consistently, Liao et al. [29] reported that liraglutide attenuated excessive microglial activation in diabetic mice brains, which decreased proinflammatory cytokine secretion and inflammatory phenotype differentiation. Further, several studies have also demonstrated that an increase in GLP-1 levels decreases the expression of the proinflammatory cytokines: TNF and IL-6, which in turn reduces the severity of inflammation [51, 52]. Other research reported that GLP-1 might be a promising therapeutic target for many neurological disorders [41].

Our research was one of the few articles to discuss the relationship between amylin and brain GSK-3β in improving cognitive disorders associated with T2DM. Also, the effect of either PRAM or LIRA on the expression of TTBK1 was not studied before. The outcomes of this research supported the neuroprotective effects of both pramlintide and liraglutide by working through several mechanisms to prevent cognitive disorders associated with DM effectively.

In conclusion, the current study supported the hypothesis that both pramlintide and liraglutide have neuroprotective effects. They were effective in protecting against HFD/STZ-induced impairment of insulin resistance markers: PI3/AKT/GSK-3β/TTBK1, also preventing the occurrence of CD. However, the LIRA group showed a slightly better significant decrease in Aβ plaque burden in the hippocampus as well as phospho-Tau-T217 expression than the PRAM group. Overall, both pramlintide and liraglutide are considered promising antidiabetic medications that may work through several mechanisms to prevent cognitive disorders associated with T2DM.

## Data Availability

Data are available upon request with the corresponding author.
