# Correction to: Liraglutide versus pramlintide in protecting against cognitive function impairment through affecting PI3K/AKT/GSK‑3β/TTBK1 pathway and decreasing Tau hyperphosphorylation in high‑fat dietstreptozocin rat model

**DOI:** 10.1007/s00424-024-02959-4

**Published:** 2024-04-05

**Authors:** Hoda M. Moghazy, Nesreen G Abdelhaliem, Sherine Ahmed Mohammed, Asmaa Hassan, Amany Abdelrahman

**Affiliations:** 1 Department of Physiology, Faculty of Medicine, Sohag University, Sohag, 82524 Egypt; 2 Department of Histology, Faculty of Medicine, Sohag University, Sohag, Egypt

**Correction to: Pflügers Archiv - European Journal of Physiology (2024)**

10.1007/s00424-024-02933-0

The original article contains an error. In the article title and Abstract, the word “TTBK” should be replaced with “TTBK1”. This is now updated here.

The original article has been corrected.

